# Atypical presentations of the sarcoidosis with kidney involvement

**DOI:** 10.12861/jrip.2012.18

**Published:** 2012-09-01

**Authors:** Hamid Nasri

**Affiliations:** ^1^Department of Nephrology, Division of Nephropathology, Isfahan University of Medical Sciences, Isfahan, Iran

**Keywords:** Sarcoidosis, Lympadenopathy, Renal failure, IgA nephropathy

**Implication for health policy/practice/research/medical education:**

The relationship between immunoglobulin A nephropathy and sarcoidosis is uncommon, however a kidney biopsy and appropriate evaluation for diagnosis of sarcoidosis can be applicable.

Previously we had reported a 68 -year-old woman with complaints of nausea, polydipsia decreased appetite, nocturia and intense constipation. The only positive finding on examination was three lymph nodes closed together in submental area. In submental lymphadenectomy, there was the multiple foci of non-caseating granulomatous reaction with multinucleated giant cells with some asteroid body, mostly consistent with sarcoidosis. The serum level of angiotensin converting enzyme was also elevated (1,2). Similar to the first case, there was a dramatic response to corticosteroid therapy and kidney failure regressed to normal value. We recently, had a 57-year-old woman, who firstly presented with hematuria. The source of hematuria was glomerular. There was also 290 mg/day proteinuria and serum calcium was 12 mg/dl. Patient also had a renal failure with serum creatinine of 1.6 mg/dl. In physical examination, the significant finding was a mild splenomegaly. Secondary work up consisting of collagen vascular tests i.e. systemic lupus erythromatosis and virus markers were negative. Chest radiography had few nonspecific infiltrations. Abdominal-CT Scan was normal except for mild splenomegaly. Serum vitamin D level and intact parathormone were normal. Bone marrow aspiration and biopsy was normal. Serum IgA level was 492 mg/dl (70-400) and a high serum level of angiotensin converting enzyme (ACE) of 82 Iu/l was detected. Also ESR was 86 mm/hr. For further evaluation a kidney biopsy was conducted. In immunofluorescence microscopy, prominent mesangial IgA deposits (#3+), in association with C_3_ deposits (#2+) and a negative C_1_q deposition was observed. In light microscopic study, a mesangial proliferation with widening of mesangial area which involved more than 50% of all glomeruli was observed (Figure 1a). There was also mesangial immune complex deposition which revealed by Masson’s trichrome stain. There was also endocapillary proliferation and hyaline thrombi (immune complex aggregation) (Figure 1b). Extracapillary proliferation was absent. Interstitial area involved by a mild fibrosis and tubular atrophy (#10%). No evidence of granulomatous inflammation was seen. However, there was focal interstitial lymphocytic infiltration too (Figure 2). The diagnosis was mostly consistent with immunoglobulin A nephropathy. According to the recent Oxford classification for immunoglobulin A nephropathy, the status of IgA nephropathy was M_1_E_1_S_1_T_1_.After one month treatment with prednisolone 1 mg/kg/day, serum creatinine and calcium regressed to 1 mg/dl and 8.5 mg/dl respectively. Serum ACE level, also decreased to normal value and after one patient is well. In this report, we aimed to provide further attention to atypical cases of sarcoidosis. We also sought to describe the rare association between sarcoidosis and immunoglobulin A nephropathy (3,4). The relationship between immunoglobulin A nephropathy and sarcoidosis is uncommon, however a kidney biopsy and appropriate evaluation for diagnosis of sarcoidosis can applicable (1-4).

Figure 1
a) Mesangial proliferation and mesangial area widening in a glomerulus in the kidney biopsy, b) Immune complex aggregation in endocapillary area.

**a:**
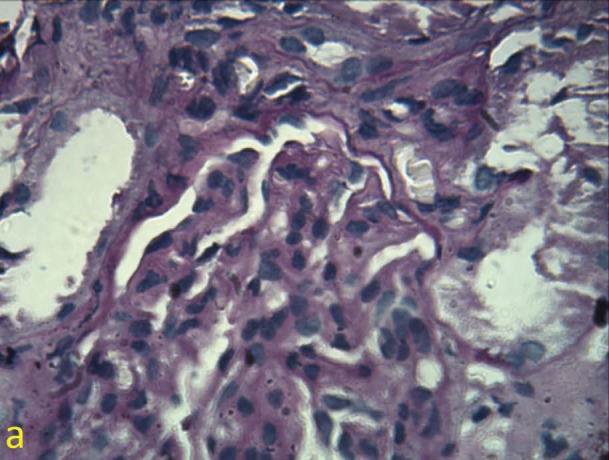

**b:**
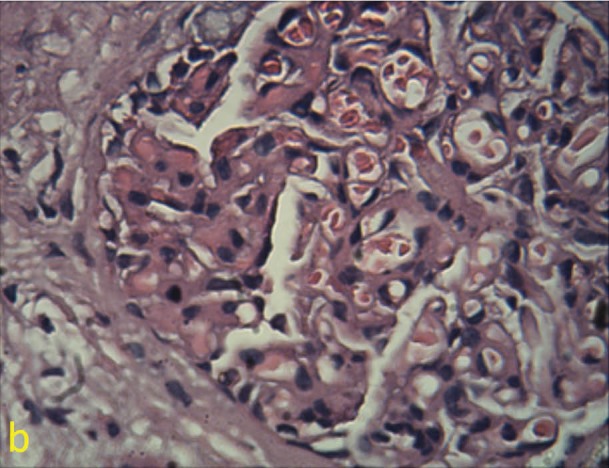

**Figure 2:**
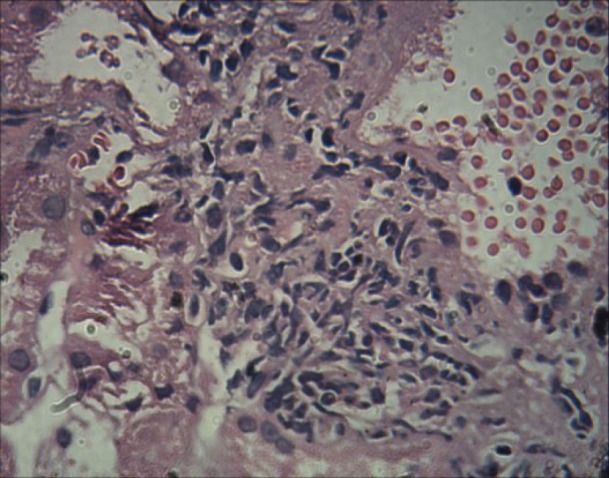
A focal infiltration in the interstitial area.

## Author’s contribution

HN is the single author of the manuscript.

## Conflict of interests

The author declared no competing interests.

## Ethical considerations

Ethical issues (including plagiarism, data fabrication, double publication) have been completely observed by the author.

## Funding/Support

None.
